# Clinical Assessment of the Efficiency of Low Level Laser Therapy in the Treatment of Oral Lichen Planus

**DOI:** 10.3889/oamjms.2015.112

**Published:** 2015-10-26

**Authors:** Hanaa M. Elshenawy, Amany Mohy Eldin, Mohamed Adel Abdelmonem

**Affiliations:** *Orodental Division Department, National Research Centre, Cairo, Egypt*

**Keywords:** Diode laser, visual analogue scale, Oral lichen planus (OLP)

## Abstract

**BACKGROUND:**

Oral lichen planus (OLP) is a chronic inflammatory disease of the oral mucosa of uncertain etiology.

**AIM:**

To evaluate the effect of using low level laser therapy (LLLT (970 nm Siro laser Advance) for the treatment of symptomatic (OLP).

**SUBJECTS AND METHODS:**

The present study was conducted on ten patients suffering from persistent oral lichen planus (OLP). Patients were treated with diode laser (970nm) for the symptomatic relief of pain and burning sensation. The patients were assessed before, during and after the completion of the laser treatment which was done twice weekly for two successive months with maximum of ten sessions. The assessment was performed using visual analogue scale (VAS) and clinical investigation for each patient.

**RESULTS:**

Detailed significant reduction in lesion size and showed complete remission of burning sensation and pain. No reported complications or therapy side effects were observed in any of the treated patients.

**CONCLUSION:**

Diode laser therapy seems to be an effective adjunctive treatment modality for relieving pain and clinical symptoms of OLP.

## Introduction

Oral lichen planus (OLP) is a relatively common chronic inflammatory disease occurs in skin and oral mucosa of unknown etiology, it rarely undergoes spontaneous remission and potentially premalignant [1]. OLP mostly affects women between fifth and sixth decade. Approximately, (18-15%) of OLP patients are smokers and (24-29%) uses alcoholic beverages [2]. Clinical subtypes have been described. The white form include reticular, popular, and plaque-like forms while the red forms include atrophic (erythematous), erosive (ulcerative) and bullous [3]. The atrophic and erosive lesions are often associated with discomfort and other symptoms [4]. The pathogenesis involves a cell-mediated immune response to antigenic changes in oral mucosa; with the predominantly T-lymphocytes infiltration [5]. Two prevalently patterns of inflammation described the plaque, and erosive type. The plaque type occurs most commonly and is estimated to affect up to 2% of population. It is present as purple raised areas with a white network known as Wickham’s striae [6]. Several empirical treatments have been used including corticosteroids, curcuminoid, oxypentifylline, as well as surgery, photochemotherapy and lasers [7]. Low level laser therapy (LLLT) recently was used for treating erosive OLP with minimal side effects [8]. LLLT is an innovative approach in medicine, which has potential biostimulating effects when applied to oral tissues, improving wound healing [9] enhances epithelization after periodontal surgery [10]. Diode lasers provide great benefits over many other lasers because of its small size comparable to other types of laser. Diode laser also provides a wide range of spectrum that may be used in medical fields ranging from physiotherapy, photodynamic therapy, and surgical uses; it is transmitted through fibrotic [11]. The physical benefit used effectively in coagulation of superficial and interstitial lesions [12]. Several low level lasers used to treat oral lichen planus, including ultraviolet waves below (350 nm length), helium-neon, more recently, the diode laser. These lasers used with different wavelengths, intensities powers, durations, number of sessions and therapeutic approaches [13].

The aim of this study was to evaluate the effect of using low level laser therapy (LLLT (970 nm Siro laser Advance) for the treatment of symptomatic (OLP).

## Subjects and Methods

The ethics committee approved the study protocol. The patients were collected from Department of Oral Medicine at the Faculty of Oral and Dental Medicine Cairo University. Ten patients with symptomatic (OLP) ranged in age from (45 to 60 years) unresponsive to topical steroids, were recruited from Dermatologic Department in Al-Qasr El Einy Hospital then referred to the Dental Clinic in National Research Center for laser treatment. Diagnosis of (OLP) is evaluated by clinical and histological features. Criteria of (OLP) diagnosis were established by World Health Organization (WHO) [14]. However, histopathological evaluation is recommended to verify clinical diagnosis and to exclude dysplasia and malignancy [15]. The selected patients had no known allergies or systemic diseases. Optimization of oral hygiene is fundamental to treat (OLP) with complete removal of supra and sub gingival plaque, whereas dental plaque and calculus implement intraoral inflammation and intensify both extensions and symptoms of (OLP) lesions. Patients were instructed to clean their teeth using a soft bristle toothbrush and toothpaste without irritants as mint or cinnamon, avoid accidental trauma on soft tissues; daily use of alcohol-free chlorhexidine mouth wash to reduce bacterial plaque, and prevent opportunistic fungal infection, especially in patients with dentures [16] throughout time of the study. Accidental toothbrush, trauma, pointed cusps, cracked teeth or worn dental restorations may worsen symptoms or may even trigger new lesions. Acidic, spicy, hard/crunchy and hot foods and beverages are not tolerated during active phases [17]. Patients with systemic disease or pregnancy were excluded. Patients were evaluated for demographics, medical history, presence of pain and discomfort, duration of disease, type, size, sites of oral involvement, these data were assessed and recorded. Patients were treated with diode laser (LLLT) 970 nm wavelength Sirolaser advance Germany). In a continuous non-contact mode with 320 µm diameter fiber optic as delivery system that directed at the affected areas of oral mucous membrane with defocused mode and overlapping exposure until blanching of the treated area was observed. The patients and the operating staff were advised to wear special googles for eye protection. Diode laser was calibrated and applied with an output power of (3W), frequency at (30 Hz), energy at (180 joule) and time interval of eight minutes divided into four subsequent sessions each of two minutes and one minute rest in between to allow for tissue relaxation. A visual analogue scale (VAS) was used to rank the severity of the patient’s symptoms ranging from zero (no pain) to ten(extreme pain) [18] and recorded before and after treatment. Clinical evaluation for the lesions was assessed and changes in the lesion’s size were monitored at the respective laser sessions. Patients were trained and motivated to maintain proper plaque control, which included teeth brushing three times a day with a soft brush and tooth paste, and rinsing once daily with alcohol-free chlorhexidine mouth wash. After each laser session, a cold diet was recommended, whereas hot and spicy food was avoided.

## Results

### Changes within each week

*Pain:* At week one, there was no statistically significant change in pain scores through all week days.

At week two, there was no statistically significant change in pain scores from Day one to Day two. From Day two to Day three, there was a statistically significant decrease in pain scores. From Day three to Day four and through the rest of week days, there was no statistically significant change in pain scores.

At week three, there was a statistically significant decrease in pain scores from Day one to Day two. From Day two to Day three and through the rest of week days, there was no statistically significant change in pain scores.

At week four, there was no statistically significant change in pain scores from Day one to Day two and from Day two to Day three. From Day three to Day four and through the rest of week days, there was no statistically significant change in pain scores.

At weeks five, six as well as seven, there was a statistically significant decrease in pain scores from Day one to Day two. From Day two to Day three and through the rest of week days, there was no statistically significant change in pain scores. While at week eight, there was no statistically significant change in pain scores through all week days.

**Table 1:** Mean, standard deviation (SD) values of pain scores at different days within each week

Week	Day 1		Day 2		Day 3		Day 4		Day 5		Day 6		Day 7		*P*-value

	Mean	SD	Mean	SD	Mean	SD	Mean	SD	Mean	SD	Mean	SD	Mean	SD	
Week 1	7.1	2.0	7.1	2.0	7.1	2.0	7.0	2.1	7.0	2.1	7.0	2.1	7.0	2.1	0.423
Week 2	6.8 ^a^	1.9	6.7 ^a^	1.9	6.4 ^b^	2.1	6.4 ^b^	2.1	6.4 ^b^	2.1	6.4 ^b^	2.1	6.4 ^b^	2.1	0.019*
Week 3	6.3 ^a^	2.2	5.9 ^b^	2.1	5.9 ^b^	2.1	5.9 ^b^	2.1	5.8 ^b^	2.0	5.8 ^b^	2.0	5.8 ^b^	2.0	0.001*
Week 4	5.3 ^a^	2.1	5.0 ^b^	1.9	4.8 ^c^	2.1	4.8 ^c^	2.1	4.7 ^c^	2.1	4.7 ^c^	2.1	4.7 ^c^	2.1	0.005*
Week 5	4.3 ^a^	2.1	4.0 ^b^	2.1	4.0 ^b^	2.1	4.0 ^b^	2.1	4.0 ^b^	2.1	4.0 ^b^	2.1	4.0 ^b^	2.1	0.006*
Week 6	3.7 ^a^	2.2	3.2 ^b^	2.0	3.2 ^b^	2.0	3.2 ^b^	2.0	3.2 ^b^	2.0	3.2 ^b^	2.0	3.2 ^b^	2.0	0.001*
Week 7	2.9 ^a^	2.1	2.4 ^b^	2.1	2.3 ^b^	2.0	2.3 ^b^	2.0	2.3 ^b^	2.0	2.3 ^b^	2.0	2.3 ^b^	2.0	<0.001*
Week 8	2.1	2.1	2.1	2.1	2.1	2.0	2.0	2.2	2.0	2.1	2.0	2.1	1.9	2.0	0.911

* *Significant at P ≤ 0.05, Different superscripts in the same row are statistically significantly different*.

### Changes through different weeks

There was a statistically significant decrease in pain scores through all weeks.

**Table 2:** Mean, standard deviation (SD) values of pain scores at different days within each week

	Mean	SD	*P*-value
Week 1	7.0 ^a^	2.0	<0.001*
Week 2	6.5 ^b^	2.1	
Week 3	5.9 ^c^	2.1	
Week 4	4.8 ^d^	2.0	
Week 5	4.0 ^e^	2.1	
Week 6	3.3 ^f^	2.1	
Week 7	2.4 ^g^	2.0	
Week 8	2.0 ^h^	2.1	

* *Significant at P ≤ 0.05, Different superscripts in the same column are statistically significantly different*.

### Lesion area

There was a statistically significant decrease in mean lesion area after treatment. The lesion area decreased by 90.0 ± 10.6% after treatment.

**Table 3:** Mean, standard deviation (SD) values and results of comparison between lesion area before and after treatment

Before treatment		After treatment		% decrease		*P*-value
Mean	SD	Mean	SD	Mean	SD	

318.2	326.2	46.0	102.8	90.0	10.6	<0.001*

* Significant at P ≤ 0.05

**Figure 1:**
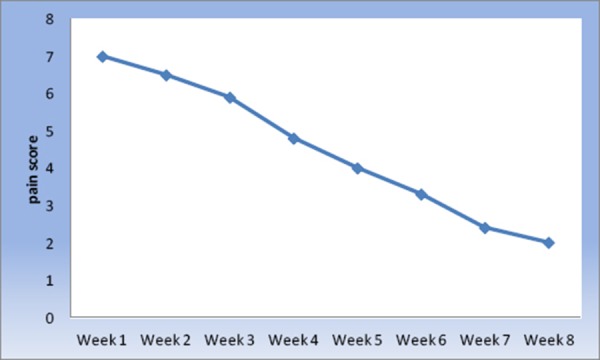
*Line chart representing mean pain scores at different weeks*.

**Figure 2:**
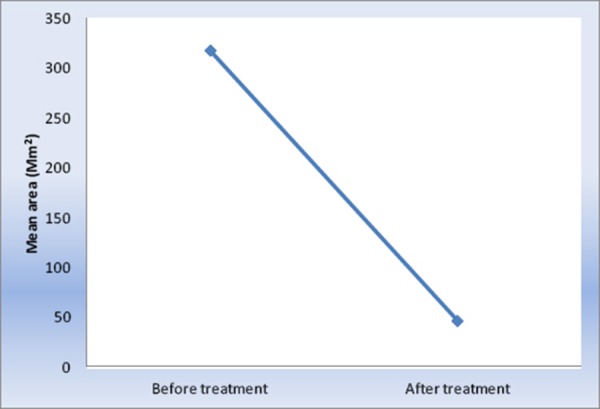
*Line chart representing mean lesion area before and after treatment*.

Initial improvement of OLP started after two weeks of treatment including the decrease in pain. Fig 1 show statistical significant decrease in pain scores starting from day two in the second week reaching its minimum score by week eight, but the patient continued to complain pain in the region of the lesion stimulated by hot and spicy food. Clinical remission was obtained in a period of two months and complete disappearance of the lesion in a period of two months is shown in Fig. 2. In this study the pain score show a high statistical improvement. Most patients reported an immediate pain relief after the second session, and all of them reported a complete resolution of symptoms at the end of the laser session, even if some lesions show partial clinical response. The results are concomitant with (Hearty, 2002) study which reported that the quality of life of patients with (OLP) improved significantly with the absence of complete resolution of all oral signs with no reported complications [19]. Cafaro et al., [20] treated 13 patients with (OLP) using the pulsed diode laser, he concluded that there was a significant decrease in lesions and decreased pain without any side effects.

## Discussion

Oral lichen planus (OLP) is a chronic inflammatory autoimmune disease characterized by remission and exacerbation. It is a cell-mediated immune condition, in which T-lymphocytes accumulate beneath the epithelium of the oral mucosa and increase rate of differentiation of stratified squamous epithelium, resulting in hyperkeratosis and erythema with or without ulceration [21]. T-cells (mostly CD8+ and CD4+ cells) migrate into the epithelium due to random encounter of antigen during routine surveillance [22]. The migrated (CD8+) cells are activated directly by antigen binding to major histocompatibility complex (MHC-1) on keratinocyte or activated (CD4+) lymphocytes [23]. The activated T-cells and amplified (Th1) cytokine production (IL-1, IL-8, IL-10, IL-12, tumor necrosis factor-alpha (TNF-α) enhancing the expression of intercellular adhesion molecule-1 (ICAM-1) on Langerhans cells and macrophages and major histocompatibility type 1 (MHC-1) antigen on keratinocytes [24]. Patients affected with erosive-atrophic lichen planus may exhibit extensive and painful lesions which are often unresponsive to conventional treatments. Corticosteroids are most widely accepted treatment for (OLP), which relieves symptoms rather than curing it [27]. Low level laser therapy (LLLT) is recent evolution on medical/dental treatments, specifically mucocutaneous lesions as (OLP) [25]. The primary benefit of (LLLT) being non-surgical, it promotes tissue healing and reduce edema, inflammation and pain. Its principle of using (LLLT) is to supply direct biostimulation light energy to body’s cells. LLLT accelerate healing, anti-inflammatory effects, increase cellular metabolism, modulation of immune system, vasodilatation and analgesic effects, and amelioration of morphine withdrawal [26]. Other modalities like calcineurin inhibitors, retinoids, dapsone, hydroxy-chloroiquine, and enoxaparin have contributed significantly towards the disease. The pathogenesis of the disease suggests that blocking (IL-12), (TNF-α), RANTES or (MMP-9) activity or upregulation (TGF-β1) activity on (OLP) may be therapeutic value in future [28]. The principle of using LLLT is to supply direct biostimulation light energy to body’s cells. Diode laser (980 nm) possesses a deep power of penetration reaching about 1.5 mm [29]. Application of diode (980 nm) will rise in temperature of affected tissues to above 50 degrees and less than 100 degrees causing protein denaturation causing blanching of treated mucosa; this denaturation means the destruction of the diseased epithelium with its surface antigen [30]. Van der Hem et al., [31] reported that re-epithelizaton occurs within three weeks after removal of the epithelium by the laser and feeling discomfort when in contact with food or liquid disappears. All laser types destroy the superficial epithelium containing target keratinocytes by protein denaturation [32]. Our results showed that the use of LLLT in the management of OLP has a significant effect on reducing pain and clinical parameters of inflammation. The initial improvement of atrophic-erosive lesions started after two weeks of treatment. Clinical remission was obtained in a period of approximately one month. Lesions that do not show any clinical improvement after four laser sessions were excluded from the study, this means that type of patient does not respond clinically, and the treatment should be stopped. The pain score had a high statistical improvement and patients successfully reported a great enhancement in quality of life with complete resolution of symptoms at the end of laser sessions. LLLT act analgesically since they improve endorphin release and therefore inhibit nociceptive signals and control pain mediators [33]. The size, site, depth, seemed to influence healing. The small number of patients diminished the possibility to draw any conclusions with statistical significance about parameters that might had more influence on the results, so further investigations with other treatment modalities and a prolonged follow-up to confirm the efficacy of laser therapy in the treatment of symptomatic OLP.
